# Cognitive insight and introspective accuracy in individuals with bipolar disorder: A scoping review

**DOI:** 10.1016/j.nsa.2023.101132

**Published:** 2023-08-19

**Authors:** Linda Wels, Nina Dalkner, Melanie Lenger, Frederike T. Fellendorf, Elena M.D. Schönthaler, Philip D. Harvey, Eva Z. Reininghaus

**Affiliations:** a Medical University of Graz, Department of Psychiatry and Psychotherapeutical Medicine, Graz, Austria; b University of Miami, Department of Psychiatry and Behavioral Sciences, Miami, USA

**Keywords:** Cognitive insight, Introspective accuracy, Bipolar disorder, Cognition

## Abstract

**Introduction:**

Within the framework of metacognition, cognitive insight (CI) refers to the ability to distance oneself from distorted beliefs and misinterpretations, and to reevaluate thoughts, beliefs, and interpretations, while also considering external feedback from others, in order to make well-considered conclusions. Similarly, introspective accuracy (IA) refers to the capacity to accurately assess one's skills, capabilities, and interpretations. CI and IA may be impaired in individuals with psychiatric disorders, as extensively documented in individuals with schizophrenia. However, there is a shortage of studies examining introspective accuracy in bipolar disorder. This scoping review aimed to provide an overview of the existing literature on CI and IA in individuals with bipolar disorder, as well as to examine their associations with clinical variables including cognitive performance.

**Methods:**

PubMed was systematically searched with the terms “cognitive insight”, “introspective accuracy”, “self estimation”, “self assessment”, “bipolar”, and “bipolar disorder”. Studies were included if they performed cognitive measures.

**Results:**

Five studies (2015–2023) met the inclusion criteria and were further analyzed. Patients with bipolar disorder generally overestimate their cognitive performance, especially in numerical tasks. In a depressive episode, the performance was underestimated and related to impaired functioning. Manic symptoms and cognitive performance have been found to be predictors for low insight. The study results indicated that introspective accuracy is positively correlated with cognitive performance.

**Conclusion:**

Recent studies demonstrate the importance of cognitive insight and introspective accuracy measures for functional outcome parameters. Although there is little research in the field of cognitive insight in bipolar disorder so far, there are many factors that still need to be addressed. Most importantly, it is needed to address the differences between the types of bipolar disorder and the impact of current mood symptoms and medication on cognitive insight or introspective accuracy in these patients.

## 1 Introduction

Two subfunctions of metacognition, cognitive insight (CI) and cognitive introspective accuracy (IA), represent aspects of people's abilities to understand and reflect upon their own mental states and processes. These two constructs differ in their focus and measurement but have recently been identified as important outcome parameters for mental illness (Gould et al., 2015). Research in psychiatric disorders concerning CI and IA has mainly focused on individuals with schizophrenia (e.g. David et al., 2012; Kao and Liu, 2010; Lincoln et al., 2007; Mervis et al., 2022; Riggs et al., 2012), yet there are not many studies that investigated this matter in individuals with bipolar disorder (BD).

Illness insight is a term used in medicine and describes the knowledge and conviction of being ill but also the understanding of how the illness affects individuals' interactions with the world (Colman, 2015). Hereby, the first step in the clinical setting is the awareness and acceptance of illness symptoms. In psychiatric diseases, patients must gain maximum knowledge and deal intensively with their symptoms to be able to develop good handling and maximum coping strategies. However, this is often difficult to achieve when symptoms distract thinking and create difficulties distinguishing real versus imagined perceptions, sensations, and thoughts which might interfere with the capability to gain illness insight (Beainy et al., 2023).

Insight can be viewed as a multidimensional construct which includes the awareness of having a psychiatric disease and the capacity to recognize associated symptoms (Crisan et al., 2018; Konstantakopoulos, 2019). Thus, insight is an important prerequisite for the acceptance of illness consequences and the need for treatment, which is related to compliance (Kemp and David, 1996; Mintz et al., 2003). However, clinical insight, which is nevertheless often limited by cognitive impairment, is often very low in individuals with mental disorders (Çetin and Aylaz, 2018; Hamilton and Roper, 2006; Zhao et al., 2015). In addition, the persistence of delusions, depressive symptoms, or psychotic symptoms might lead to misinterpretations or to neglecting corrective feedback, thus impairing perception of symptoms and insight into disease (Beainy et al., 2023; Mintz et al., 2003). According to the literature, there is an impairment in clinical insight, delusional beliefs, and thinking in many patients with mental illnesses which might be linked to cognitive impairment (Li et al., 2020; Stratton et al., 2013; Subotnik et al., 2020). Cognitive deficits are often decisive reasons why psychiatric patients have difficulties in everyday and professional life (Elias et al., 2017; Fatouros-Bergman et al., 2014; Kharawala et al., 2022; Rock et al., 2014; Samamé et al., 2014; Semkovska et al., 2019). Therefore, it is important that individuals with psychiatric disorders are supported in receiving awareness of their illness symptoms, attributing the symptoms to the disease, and achieving compliance with treatment to increase their quality of life (David, 1990; Davis et al., 2020). Paradoxically, good clinical insight as well as CI has also been found to hinder health. Some studies suggested that the awareness of illness was associated with depressive symptoms or self-stigma (Amore et al., 2020; Belvederi Murri et al., 2015; Mervis et al., 2022). This phenomenon has been termed the “insight paradox” and possibly results from the realization of the implications and consequences of suffering from a chronic illness. Among others, recovery attitudes, illness severity, rumination, internalized stigma, illness perception, socioeconomic status, and premorbid adjustment have been suggested as moderators and/or mediators of the association between insight and depression (Belvederi Murri et al., 2015, 2016).

More specifically and differing from clinical insight, CI is the ability to re-evaluate thoughts, beliefs, and interpretations to make thoughtful conclusions (Beck et al., 2004a). In detail, CI is the ability to recognize one's own thought patterns, reasoning styles, biases, and assumptions that may be influencing one's behavior or decision-making (Jørgensen et al., 2015; Riggs et al., 2012). Thus, CI is one aspect of metacognitive function (Lysaker et al., 2008) referring to the ability to assume perspective about misinterpretations and to reassess those (Brune et al., 2011). It involves a deeper understanding of one's mental processes and how they relate to the situation at hand (van der Werf-Eldering et al., 2011). Importantly, CI also includes external feedback from others to gain better self-reflectiveness and self-certainty (Riggs et al., 2012). Individuals with higher self-reflectiveness are able to consider different perspectives and evaluate alternative hypotheses before concluding while self-certainty reflects the personal conviction of the accuracy of the belief (Beck et al., 2004a). CI is often measured through self-report questionnaires, such as the Beck Cognitive Insight Scale (BCIS; Beck et al., 2004b) or the Metacognition Assessment Scale - Abbreviated (MAS-A; Bröcker et al., 2017).

In addition to CI, which is focusing on beliefs and thoughts, also cognitive abilities can be consciously or unconsciously. The accuracy of one's own self-assessment of cognitive performance and actual cognitive performance is called IA and has been measured in recent studies by asking participants to judge their test scores immediately after completion (Harvey and Pinkham, 2015) The self-estimation is compared with the objective test score. Usually, the results are correlated with each other. A positive correlation indicates good IA, a negative correlation indicates poor IA. An introspective bias, for example overestimation, occurs when the subjective score is much higher than the objective test score. In previous studies, IA has been determined as an important independent predictor of everyday functioning (Harvey and Pinkham, 2015; Silberstein and Harvey, 2019) as well associal and vocational functioning (Gould et al., 2015).

Research in psychiatric disorders concerning clinical insight and CI, as well as IA, has mainly focused on individuals with schizophrenia. Individuals with symptoms of psychosis are strongly limited in their capacity to review their thinking problems, recognize their errors, and correct them (Riggs et al., 2012). Furthermore, low clinical insight and CI, as well as misestimation of performance and being more confident in their beliefs (measured through self-certainty with the BCIS) have been described (David et al., 2012; Kao and Liu, 2010; Lincoln et al., 2007). In a recent review, higher levels of CI and IA have been associated with better community functioning and neurocognition in individuals with schizophrenia However, higher CI was also linked to increased levels of suicidality (Mervis et al., 2022).

Regarding other psychiatric diseases, there is less research. There are a few studies (e.g., Buchy et al., 2009; Colis et al., 2006) investigating the association between anxiety and CI, but without consistent results. Another study investigated CI in obsessive-compulsive disorder found that the BCIS score did not correlate with other measures used to observe clinical insight (Shimshoni et al., 2011). In individuals with Alzheimer's disease, both self-reflectiveness and self-certainty were significantly lower compared to healthy controls (Degirmenci et al., 2013). In individuals with Parkinson's disease, there is a study comparing patients with and without an impulse control disorder. CI and self-reflectiveness were shown to be greater in individuals with high impulsivity (Mack et al., 2013).

In individuals with BD, awareness of illness, CI, and IA might be highly variable depending on the current state of mood (Cassidy, 2010; Dell'Osso et al., 2000). BD is a chronic mood disorder with pathological mood episodes ranging from depression to mania, with a lifetime prevalence of 1.06% (BD type I) respectively 1.57% (BD type II). The one-year prevalence for BD type I was 0.71%, and for BD type II it was 0.50% (Clemente et al., 2015). In individuals with BD, everyday functioning is limited, as patients often spend time alone and at home, work less, and spend more time in passive activities (Havermans et al., 2007). Acute mania is often characterized by a lack of insight, while in depression, patients often suffer more and have more illness insight during acute episodes. In euthymia, individuals with BD have probably the highest chances to achieve knowledge about their illness, as well as to learn to recognize the symptoms of disease at an early stage (Crisan et al., 2018).

There are not many studies that investigated a possible connection between CI/IA, and cognitive performance as well as functioning especially in individuals with BD. The aim of this scoping review was therefore to summarize the existing literature on CI and IA in individuals with BD and their correlation with clinical and illness-related symptoms such as cognition and functioning.

## 2 Method

### 2.1 Information sources and search strategy

A systematic literature search and selection for peer-reviewed articles was conducted by L.W. and E.M.D.S. using PubMed. There occurred no conflicts.

The search strategy included the keywords: “cognitive insight”, “introspective accuracy”, “self estimation” and “self assessment” combined with “bipolar disorder” or “bipolar”. The following filters were applied: for text availability, the filter “full text” was applied, for article type the filters “Clinical Trial”, “Meta-Analysis” and “Randomized Controlled Trial” were applied and only studies published in the last 20 years (01.01.2003–14.04.2023) were included.

### 2.2 Study selection process

Inclusion criteria required (1) studies investigating adult humans with the diagnosis of BD assessed by the Diagnostic and Statistical Manual of Mental Disorders (DSM) or the International Classification of Diseases (ICD) criteria (all versions); (2) the use of cognitive performance tests without specification on a certain domain, (3) original data in an observational design, including retrospective or longitudinal prospective studies, and (4) studies written in English. Case reports and conference papers were excluded. All publications possibly fulfilling eligibility criteria were retrieved for review of the manuscript.

## 3 Results

**Fig. 1:**
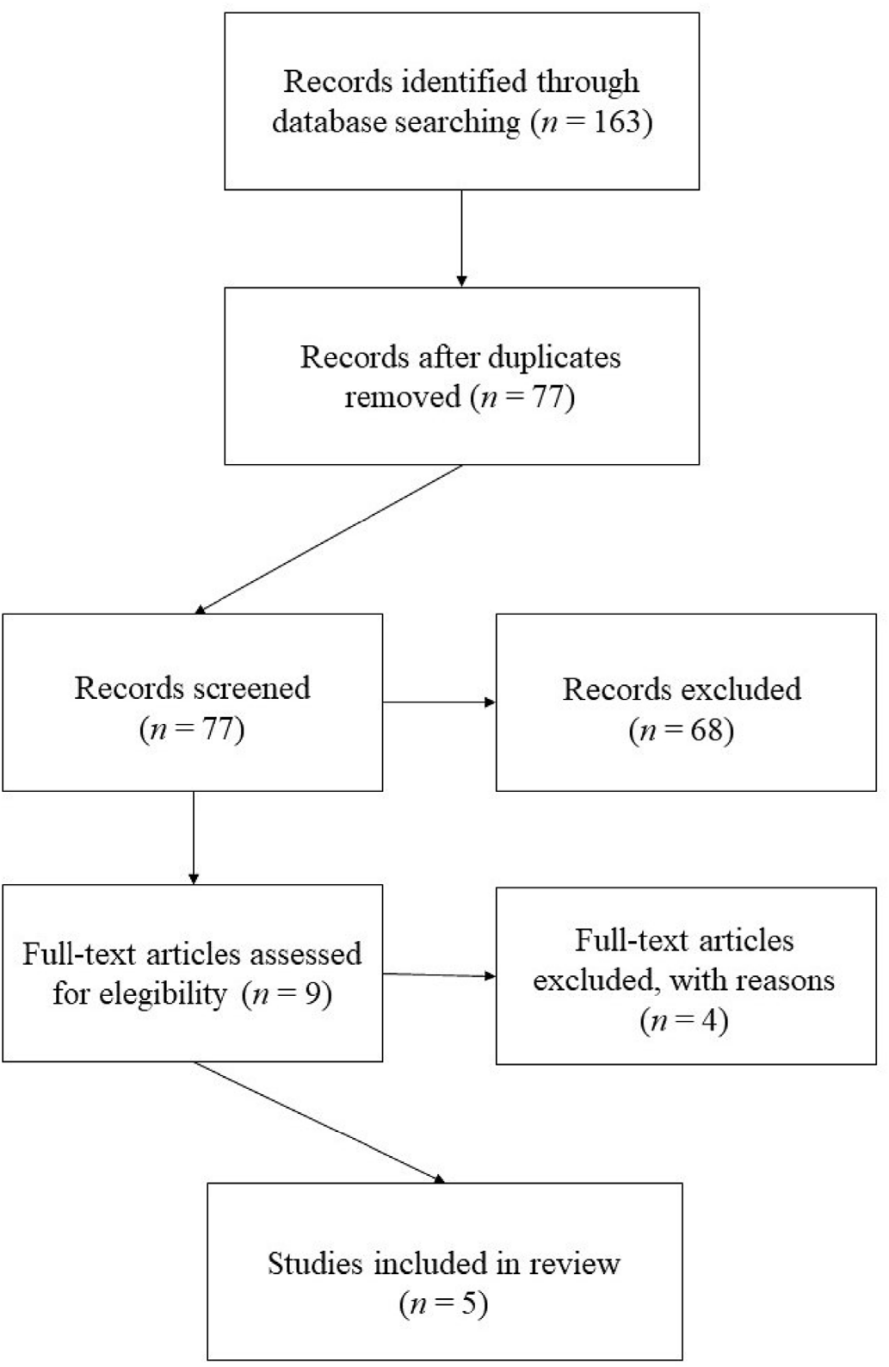
Study inclusion process of the databases PubMed Note: Reasons for exclusion: Records excluded: 68 articles did not investigate BD or meet the inclusion criteria; Full-text articles excluded, with reasons: Two studies did not include cognitive tests, two studies did not investigate CI or IA.

A PRISMA flowchart (Page et al., 2021) describes the screening procedure for the retrieved records (Fig. 1). Five publications (Camelo et al., 2019; Dalkner et al., 2023; Harvey et al., 2015; Morgan et al., 2022; Tercero et al., 2021) met the inclusion criteria and were further analyzed. The review includes one publication on the keywords “cognitive insight bipolar disorder”, zero publications on the keywords “cognitive insight bipolar”, two publications on the keywords “introspective accuracy bipolar disorder”, zero publications on the keywords “introspective accuracy bipolar”, zero publications on the keywords “self estimation bipolar disorder”, zero publications on the keywords “self estimation bipolar”, two publications on the keywords “self assessment bipolar disorder” and zero publications on the keywords “self assessment bipolar”. Table 1 presents an overview of the included studies, the clinical variables including cognitive assessments, as well as sample size, study design, and main results. Two of the explored papers (Dalkner et al., 2023; Morgan et al., 2022) investigated an overlapping sample. Nevertheless, we included both samples, as they investigated different questions.

**Table 1:** Sample characteristics, assessments, and findings for included studies (N ​= ​5).

Authors, Year	Sample size and sample description	Study design	Cognitive domain	Cognitive tests	Results in individuals with BD on CI	Results in individuals with BD on IA
Camelo et al. (2019)	*N* ​= ​65 BD (*N* ​= ​65; 34 in euthymia, 11 in manic episode, 20 in depression episode)	Cross-sectional study	Verbal short-term and working memory Processing of visual symbols Inhibition Speed of Processing Verbal fluency	DGS & Letter-Number Sequencing (from WAIS-R) Symbol Search (from WAIS-R) SCWT TMT-A & TMT-B Verbal fluency test (from MEC B)	Individuals during mania: poorer global insight and insight about treatment effectiveness. Loss of insight can be predicted with severity of manic symptoms and impairments in processing of visual symbols (Symbol Search Test), speed of processing (TMT) and inhibition (SCWT).	
Dalkner et al. (2023)	*N* ​= ​240 BD (*N* ​= ​114) Schizophrenia or schizoaffective disorder (*N* ​= ​126)	Longitudinal study (30 days)	Speed of Processing Semantic fluency Working Memory Verbal Learning	TMT-A (from MCCB) ANT (from MCCB) Letter-Number Sequencing (from MCCB) HVLT-R (from MCCB)		Performance was overestimated in numerical tasks. Higher levels of predominant negative affect over 30 days were associated with lower IA.

Authors, Year	Sample size and sample description	Study design	Cognitive domain	Cognitive tests	Results in individuals with BD on CI	Results in individuals with BD on IA
Harvey et al. (2015)	*N* ​= ​30 BD type I (*N* ​= ​30)	Cross-sectional study	Speed of Processing Semantic fluency Verbal and Learning Reasoning and Problem-solving Attention/Vigilance Attention, working memory, and visual processing Functional Capacity and assesses skills Social functioning Cognitive impairment	TMT-A & Symbol Coding (from MCCB) ANT (from MCCB) HVLT-R (from MCCB) NAB – Mazes Subtest (from MCCB) CPT-IP (from MCCB) Letter-Number Sequencing, WMS-III–SSP & BVMT-R (from MCCB) & Metacognitive WCST UPSA-B SLOF CAI		Individuals in depressive episodes underestimated their cognitive and independent living skills. Self-reported depressive symptoms correlated with more impaired functioning.
Morgan et al. (2022)	*N* ​= ​321 Schizophrenia (*N* ​= ​163) BD (*N* ​= ​158)	Longitudinal study (30 days)	Speed of Processing Semantic fluency Working Memory Verbal Learning	TMT-A & Symbol Coding (from MCCB) ANT (from MCCB) Letter-Number Sequencing (from MCCB) HVLT-R (from MCCB)		Poorer cognitive performance had a significant covariate effect on absolute IA Better cognitive performance was associated with better IA.

Authors, Year	Sample size and sample description	Study design	Cognitive domain	Cognitive tests	Results in individuals with BD on CI	Results in individuals with BD on IA
Tercero et al. (2021)	*N* ​= ​166 Schizophrenia (*N* ​= ​99) BD (*N* ​= ​67)	Cross-sectional study	Attention, working memory, and visual processing	Metacognitive WCST		Performance was overestimated on the WCST by about 50%. WCST was the best predictor of the global judgements compared to trial-by-trial accuracy judgments and trial-by-trial confidence ratings. More overestimation of performance in the WCST was associated with poorer global judgement of performance.

Note: ANT ​= ​Animal Naming Test, BVMT-R = Brief Visuospatial Memory Test – Revised, CAI = Cognitive Assessment Inventory (Ventura et al., 2013), CI = Cognitive Insight, CPT-IP = Continuous Performance Test—Identical Pairs, DGS ​= ​Digit Span, HVLT-R = Hopkins Verbal Learning Test—Revised, IA = Introspective Accuracy, MCCB ​= ​MATRICS Consensus Cognitive Battery (Nuechterlein et al., 2008), MEC B = Montreal Communication Evaluation Battery (Fonseca et al., 2008), NAB = Neuropsychological Assessment Battery, SCWT = Stroop Color and Word Test (Stroop, 1935), SLOF = Specific Levels of Functioning (Schneider and Struening, 1983), SSP = Spatial Span Test, TMT-A ​= ​Trail Making Test – Part A (Gaudino et al., 1995), TMT-B ​= ​Trail Making Test – Part B (Gaudino et al., 1995), UPSA-B Performance-based Skills Assessment (Mausbach et al., 2007), WAIS-R = Wechsler Adult Intelligence Scale-Revised (Wechsler, 1955), WCST = Wisconsin Card Sorting Test (Koren et al., 2004), WMS-III = Wechsler Memory Scale – 3rd edition.

Camelo et al. (2019) investigated 65 individuals with BD and found that patients in acute mania showed poorer global insight and insight about treatment effectiveness compared to patients with depressive episodes or euthymia. The authors suggested that the severity of manic symptoms and cognitive performance can be strong predictors of loss of insight in individuals with BD. Together with poor performance in cognitive tasks in working memory and processing speed, manic symptoms could predict CI.

In a recent study by Dalkner et al. (2023) investigating 114 patients with BD (type I and II), a relationship between predominant negative affect over a 30-day sampling period and poor IA has been observed. In schizophrenia higher negative affect predicted lower absolute misestimation. Cognitive performance was overestimated in the observed groups in numerical tasks.

Harvey et al. (2015) examined 30 patients with a lifetime history of BD type I and observed an association between depressive symptoms, IA, everyday functioning, and clinician ratings. This study found that individuals with BD underestimated their cognitive and independent living skills during a depressive episode. In contrast, clinicians rated cognitive abilities and living skills higher than the patients themselves.

Morgan et al. (2022) demonstrated that individuals with BD showed better cognitive performance than individuals with schizophrenia. In both groups, better cognitive performance was associated with better IA. Poorer cognitive performance was shown to have a significant covariate effect on absolute IA.

A study by Tercero et al. (2021) investigating performance and judgments of performance, using the Wisconsin Card Sorting Test (WCST) in 67 individuals with BD, found that these individuals overestimated their performance by about 50%. Regression analyses showed that performance in the WCST performance is a better predictor of the global judgment for BD than trial-by-trial accuracy judgments and trial-by-trial confidence ratings. Apparently, patients with BD incorporated the feedback that was supplied in the metacognitive WCST, whereas patients with schizophrenia did not. The more patients with BD overestimated their performance, the poorer their global judgment of their performance was.

## 4 Discussion

The aim of this scoping review was to give an overview of CI and IA in individuals with BD, and to show associations between CI and IA with clinical parameters e.g., depressive symptoms, neurocognitive performance, and functioning.

So far, not many studies exist investigating CI and IA and their associations with clinical symptoms and functioning in BD. However, preliminary findings indicate that both CI and IA appear to play a significant role in predicting the trajectory of illness and everyday functioning in individuals with BD (e.g., Harvey et al., 2015; Henry et al., 2013). The majority of studies emphasized the clinical importance of individuals' subjective perceptions of their cognitive abilities, rather than their actual performance on cognitive tests. Burdick et al. (2005) were among the pioneers in examining self-assessment of cognitive performance in BD, although their terminology differed from that used in later studies (“introspective accuracy”). Their study revealed that the self-report questionnaires did not correlate with mood ratings of mania or depression, nor did they provide accurate predictions of neuropsychological impairment. Accordingly, most existing studies found that individuals with BD did not accurately assess their cognitive performance and that most of these individuals overestimated their cognitive performance, especially in numeric tasks (Burdick et al., 2005; Dalkner et al., 2023; Tercero et al., 2021). However, during a depressive episode, individuals with BD tend to underestimate their cognitive skills (Harvey et al., 2010). Healthy controls typically demonstrate better estimation skills in cognitive tests (Fragkiadaki et al., 2016), although this effect appears inconsistently across the general population.

Overall, the findings highlight that CI and IA in BD are not stable traits but rather state markers that can vary significantly across illness trajectories. An individual's self-assessment of their cognitive abilities can fluctuate, leading to either overestimation or underestimation depending on their current affective state. This variability suggests that IA is influenced by the individual's emotional state at the time of assessment, rather than being a consistent characteristic or trait. In accordance, there is evidence that individuals in a manic episode have less clinical insight including less insight into treatment effectiveness (Cassidy, 2010). Of the examined studies, only one differentiated between the phases of individuals with BD (Camelo et al., 2019). There was no difference found in cognitive performance when comparing the episodes. This raises the important question of how the varying levels of CI and IA throughout the course of BD can have differential impacts on individuals' everyday lives and functioning. Further investigation is warranted to explore this aspect in detail. Furthermore, it is crucial to examine potential differences in CI and IA between different subtypes of BD, such as BD type I and BD type II, as well as subclinical BD. Future research should aim to shed light on these distinctions to enhance our understanding of the disorder and its manifestations.

During our research, two studies were excluded because they did not examine CI or IA as defined above. Nevertheless, these studies provided relevant information, as they investigated the influence of cognitive training on cognition in individuals with BD. It was shown that ten and twelve weeks of cognitive training have a positive impact on executive functioning, working memory, and self-efficacy in BD (Ott et al., 2021; Twamley et al., 2019). As executive functioning, working memory, and self-efficacy are limited in individuals with BD (Abraham et al., 2014; Cotrena et al., 2020), cognitive training could help in improving those. In patients with psychosis, executive function and working memory are positively associated with insight (Nair et al., 2014). Cognitive training could improve those and therefore insight. Possibly, there is a way to not only train cognition in individuals with BD but also the related insight and introspection, which could be administered and examined in future studies.

In the present investigation, we were also interested in the correlates of CI and IA in BD. One study on schizophrenia suggested that impaired CI depends on reduced working memory and executive function capacity (Orfei et al., 2010). Weiler et al. (2000) found a correlation between psychopathology and insight, emphasizing that insight in patients with BD is particularly influenced by psychopathology, and more so than in individuals with schizophrenia or schizoaffective disorder. This was further supported by the results by Dalkner et al. (2023), who demonstrated that negative affect is more strongly associated with IA in individuals with BD compared to those with schizophrenia.

Individuals with BD and schizophrenia share impairments in many cognitive domains, however, patients with BD typically show less severe deficits (Bora et al., 2010; Harvey et al., 2010; Lynham et al., 2018). More research is needed to shed more light on transdiagnostic CI and IA differences to fully understand the mechanisms of these cognitive processes and thus help individuals with BD to compensate for potential deficits. Subsequently, cognitive training and treatment options to prevent deterioration in CI and IA could be established and examined.

Several studies provide support for the curvilinear relationship between cognitive ability and insight. These results suggest that the connection between insight and cognition is intricate, potentially involving interactions between cognitive abilities and other factors (Cooke et al., 2007). However, it is important to note that these findings do not specifically address individuals with BD, indicating a need for further exploration in this area.

It is important to note that the studies reviewed did not assess patient medication. There is research examining the effects of pharmacological treatments on cognition in BD (e.g., Xu et al., 2020; Young et al., 2004), but no studies were found that specifically examined the effects of medication on CI and IA in individuals with BD; this should certainly be considered in future studies. Accordingly, there is little to no research regarding the neurobiological foundations of CI and IA. A study by Riggs et al. (2012) indicated that the development of clinical insight depends on a certain degree of CI. We suggest that future research should investigate the neurobiological foundation of low CI and IA in BD, and the effects of CI on treatment response. In addition, longitudinal studies with high sample sizes are highly needed to establish the stability of CI and IA and the predictive value of CI and IA on functional long-term outcome parameters.

Comparing CI and IA in patients with BD and healthy controls could help to understand the illness-specific aspects of CI and IA and the contribution of CI/IA to cognitive and functional impairments in patients with BD. A longitudinal study investigating cognitive function and associations with CI/IA in individuals with BD in dependence of bipolar subtypes and illness characteristics would increase knowledge in metacognitive research of BD.

Based on our clinical experience with patients and the first evidence from the literature, we have recognized the significance of CI and IA in BD, prompting us to conduct an initial overview to shed light on these topics and introduce them to the field. In our literature assessment, we utilized keywords including “cognitive insight,” “introspective accuracy,” “self-estimation,” and “self-assessment” in conjunction with “bipolar disorder” or “bipolar” to evaluate the existing literature. However, as we delved deeper into the subject, we discovered numerous articles that explored CI and IA but employed different terminology to describe these constructs. Examples are “patient-evaluated cognitive function” (Faurholt-Jepsen et al., 2020), “subjective cognitive function” (Demant et al., 2015), “subjective complaints” (Martinez-Aran et al., 2005), “subjectively reported cognitive functioning” (Svendsen et al., 2012), and “cognitive complaints” (van der Werf-Eldering et al., 2011). Therefore, future reviews on this topic should expand their keyword selection to include articles using alternative terms. Given that CI and IA are relatively new areas of investigation, there is currently no consistent definition or standardized measurement for these constructs.

In conclusion, CI and IA are important metacognitive functions related to our ability to understand and reflect upon our own thoughts, emotions, beliefs, and abilities. Consistently, the few studies that exist show that better insight into cognitive abilities is associated with better functioning in BD, and should therefore be targeted in treatments. Additionally, it should be emphasized that CI and IA exhibit considerable variability within individuals diagnosed with BD, depending on their affective state. However, there is limited research examining the longitudinal nature of CI/IA and its potential biological correlates in BD. Therefore, further research is needed to fully explore these aspects and improve our understanding of the complex interplay between CI/IA, functioning, and underlying biological mechanisms.

## Declaration of competing interest

The authors declare that they have no known competing financial interests or personal relationships that could have appeared to influence the work reported in this paper.
